# Robotic Cell Rotation Based on Optimal Poking Direction

**DOI:** 10.3390/mi9040141

**Published:** 2018-03-22

**Authors:** Chunlin Zhao, Yaowei Liu, Mingzhu Sun, Xin Zhao

**Affiliations:** 1 Institute of Robotics and Automatic Information System (IRAIS), Nankai University, Jinnan District, Tianjin 300350, China; zhaocl@mail.nankai.edu.cn (C.Z.); nkliuyaowei@foxmail.com (Y.L); zhaoxin@nankai.edu.cn (X.Z.); 2 Tianjin Key Laboratory of Intelligent Robotics (TJKLIR), Nankai University, Jinnan District, Tianjin 300350, China

**Keywords:** robotic cell rotation, optimal poking direction, slip-resistance, high efficiency

## Abstract

It is essential to have three-dimensional orientation of cells under a microscope for biological manipulation. Conventional manual cell manipulation is highly dependent on the operator’s experience. It has some problems of low repeatability, low efficiency, and contamination. The current popular robotic method uses an injection micropipette to rotate cells. However, the optimal poking direction of the injection micropipette has not been established. In this paper, a strategy of robotic cell rotation based on optimal poking direction is proposed to move the specific structure of the cell to the desired orientation. First, analysis of the force applied to the cell during rotation was done to find the optimal poking direction, where we had the biggest moment of force. Then, the moving trajectory of the injection micropipette was designed to exert rotation force based on optimal poking direction. Finally, the strategy was applied to oocyte rotation in nuclear transfer. Experimental results show that the average completion time was up to 23.6 s and the success rate was 93.3% when the moving speed of the injection micropipette was 100 μm/s, which demonstrates that our strategy could overcome slippage effectively and with high efficiency.

## 1. Introduction

It is necessary to adjust cells or move specific structures of cells to the desired orientation before biological manipulation, such as in nuclear transfer [1,2,3] or zebrafish embryo injection [4,5,6]. Taking nuclear transfer as an example, the polar body of the oocyte needs be rotated to the desired orientation, such as 2 o’clock or 4 o’clock, before enucleation. At present, most biological manipulations are done manually, and these manual manipulations demand a high professional level of operators. With the development of robotics and automatic techniques, a number of minimally invasive techniques have been used to adjust the position and orientation of the cell.

Some biomanipulation methods based on hydrodynamic force have been proposed [7,8]. Yalikun put forward a method using hydrodynamic rotation for a single cell [9]. However, using this method, it is not convenient to hold the cell for nuclear transfer. Microfluidic technology has also attracted the interest of researchers. Leung applied microfluidic flow control to perform 3-D rotation of mouse oocytes [10]. However, this method is not suitable for manipulating batch oocytes and the efficiency is low. Wang demonstrated a technique that utilized the friction force exerted on the standard micropipette and microchannel to rotate the embryo to a desired orientation [11]. However, this method is not suitable for small cells. A method based on dielectrophoresis is widely used [12,13,14,15]. However, the increased electrical field may affect the properties of the cell membrane and lead to potential damage when the voltage of the electrical field exceeds a certain range. The use of optical tweezers is a technique that applies optical forces to manipulate the cell [16,17,18,19,20]. However, the accuracy of optical tweezers is limited due to the easy capture of any dielectric particles near the laser trap. Zhao put forward an approach of robotic cell rotation based on minimum rotation force [21]. The rotation force exerted on the cell was related to the deformation of the cell when the injection micropipette poked it. However, the efficiency was low because the optimal poking direction of the injection micropipette based on minimum rotation force was not used.

To solve these problems, we calculated the optimal poking direction of the injection micropipette to have the biggest moment of force. The biggest moment of force leads to the highest efficiency when the same force is exerted on the cell by the injection micropipette. Furthermore, we propose a strategy of robotic cell rotation based on the optimal poking direction of the injection micropipette. We applied this strategy to oocyte rotation in nuclear transfer. The experimental results demonstrate the system’s capability for fast oocyte orientation and high efficiency.

The rest of the paper is organized as follows: First, we analyze the force of cell rotation and find the optimal poking direction of the injection micropipette in Section 2.1. Then, the strategy of robotic cell rotation based on optimal poking direction is introduced in Section 2.2. The experiments on porcine oocytes, designed to validate the feasibility of the strategy, are described in Section 3.1 and Section 3.2. After that, the experimental results are given in Section 3.3. Finally, some discussions are given in Section 4.

## 2. Materials and Methods 

### 2.1. Derivation of Optimal Poking Direction

**Figure 1:**
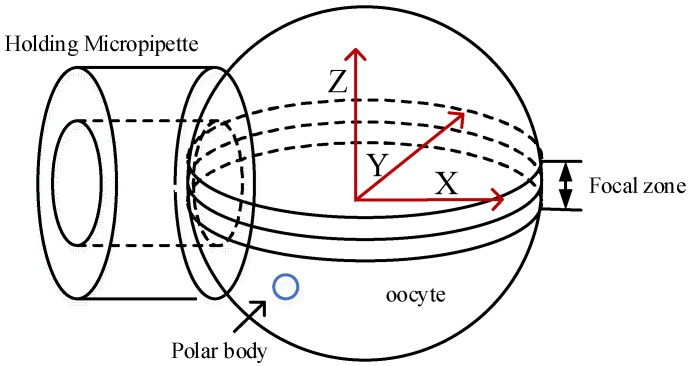
Three-dimensional model of oocyte rotation.

Figure 1 illustrates the three-dimensional model of oocyte rotation. During the process of cell rotation, the injection micropipette and holding micropipette are used to revolve and hold the oocyte, respectively. Because of occlusion of the cytoplasm, which is not transparent in the bright field of the microscope, the polar body can be observed only when it is revolved into an area near the focal plane. This area was defined as the focal zone. Therefore, on the basis of whether the polar body could be observed, 3-D cell rotation could be decoupled into two plane motion processes, the revolving course and the rotation course. We defined *O*-*XYZ* as the cell coordinate frame, where *O* is located at the center of the cell. The X axis followed the central axis of the holding micropipette, and the *X*-*Y* plane was consistent with the focal plane. The revolving course (the first stage) was described as the process of moving the polar body to the focal zone (near the *X*-*Y* plane), rotating the cell around the *Y* axis. In the rotation course (the second stage), the polar body was moved to the desired angular orientation, such as 11 o’clock, and the cell was rotated around the *Z* axis.

Through many previous experiments, we found that the poking position of the injection micropipette could not revolve the cell normally when the poking position was the intersection of the cell membrane and the *X* axis. To explain this phenomenon, we analyzed the forces of cell rotation in the rotation course. The rotation speed of the cell was very low (second level) and the cell could be seen in an equilibrium state. According to the principle of rigidization, the cell can be assumed to be in rigid body mode, and an analysis of static theory can be performed to calculate the optimal poking position. In addition, the direction of rotation of the cell was assumed to be counterclockwise.

As mentioned above, first we carried out the corresponding mechanical analysis in the rotation course where the injection micropipette runs in the *X*-*Y* plane and makes the oocyte rotate around the *Z* axis. Then the same analysis was applied to the revolving course.

**Figure 2:**
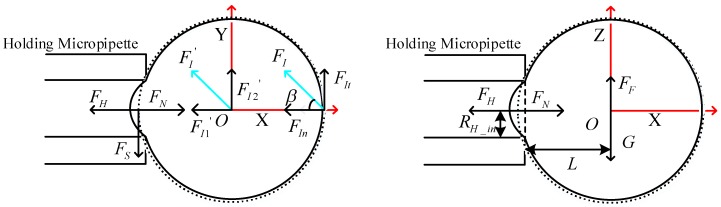
Initial poking direction of injection micropipette in the *x* axis.

Figure 2 depicts the schematic of forces in the rotation course. The rotation force *F_I_* is exerted by the injection micropipette. *F_I_* can be divided into normal force *F_In_* along the center of the cell and tangential force *F_It_* along the tangential direction. The inclined angle of *F_I_* and *F_In_* is defined as *β*. Their relationship is shown as:

(1) $$\cos\beta =F_{In}/F_{I}$$

(2) $$F_{I}=\sqrt{F_{In}{}^{2}+F_{It}{}^{2}}$$

The effects exerted by the holding micropipette can be divided into the holding pressure *F_H_*, the static fiction force *F_S_*, which is parallel to the negative direction of the *Y* axis, and the supporting force *F_N_* along the *X* axis. The resisting force between the cell and the culture media was in accordance with Newton’s law of inner friction. As the cell was in an equilibrium state, the fluid velocity gradient du/dy was approximately equal to zero, according to Newton’s law of inner friction, F=μA(du/dy). Therefore, the resisting force between the cell and the culture media could be ignored, and the main resisting force was the force between the cell and the holding micropipette.

When we carried out the corresponding mechanical analysis in the revolving course, where the injection micropipette runs in the *X*-*Z* plane, in order to balance the effects of gravity *G* and buoyancy *F_F_*, the effects of the holding micropipette exerted on the cell can also be divided into the force along the *X* axis and the force along the *Z* axis.

On the condition that the cell is in the equilibrium state, the impact of *F_I_* can be replaced by a force *F^’^_I_* and a rotation moment *m* at point *O*, where *F^’^_I_* is equal to *F_I_*. Further, *F^’^_I_* can be divided into *F^’^_I_*_1_ along the *X* axis and *F^’^_I_*_2_ along the *Y* axis. They have a relationship as follows:

(3) $${F^{\prime }}_{I1}+F_{H}=F_{N}+L*(G-F_{F})/R_{H\_in}$$

(4) $${F^{\prime }}_{I2}=F_{S}$$

where *R_H_in_* is the inner radius of the holding micropipette and *L* is the distance from the center of the holding micropipette to *O*, which can be expressed as:

(5) $$L=\sqrt{R^{2}-R_{H\_in}{}^{2}}$$

where *R* is the radius of the cell.

As the cell is rotated counterclockwise, the static fiction force *F_S_* should be parallel to the positive direction of the *Y* axis. This conclusion conflicts with the above assumption; the injection micropipette cannot revolve the cell normally in this poking position and direction. Therefore, the poking direction of the injection micropipette is estimated to relate to the inclined angle *β* and be parallel to the *X* axis in the *X*-*Y* plane.

**Figure 3:**
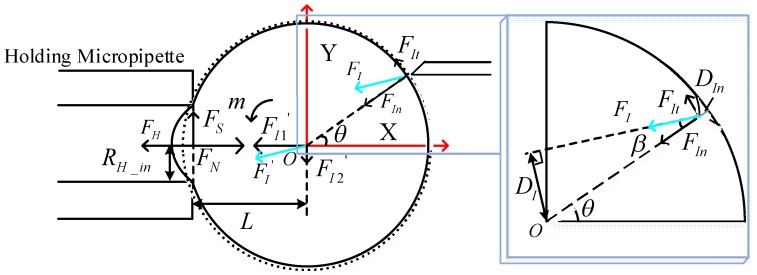
Initial poking position of injection micropipette perpendicular to the *X* axis.

As shown in Figure 3, *F^’^_I_* can also be divided into *F^’^_I_*_1_ along the *X* axis and *F^’^_I_*_2_ along the *Y* axis. They are derived as:

(6) $${F^{\prime }}_{I1}={F^{\prime }}_{I}*\cos(\theta -\beta )$$

(7) $${F^{\prime }}_{I2}={F^{\prime }}_{I}*\sin(\theta -\beta )$$

where *θ* is the angular orientation of the contact point between the injection micropipette and the *X* axis (θ≥β). The rotation moment *m* at *O* in the *X-Y* plane can be derived as:

(8) $$m=F_{I}*D_{I}-F_{S}*L=F_{I}*(R-D_{In})*\sin\beta -F_{S}*L$$

where *D_In_* is the poking depth, and the relationship of *D_I_* and *D_In_* is $D_{I}=(R-D_{In})*\sin\beta$. Substituting Equations (4), (5), and (7) into (8), *m* can be calculated as:

(9) $$m=F_{I}*(R-D_{In})*\sin\beta -F_{I}*\sin(\theta -\beta )*\sqrt{R^{2}-R_{H\_in}{}^{2}}$$

If the magnitude and direction of the rotation force *F_I_* are constant, the optimal initial poking direction should make the rotation moment *m* largest. Since *F_I_*, *R_H_in_*, *D_In_*, *R*, *β* are constant, the different poking positions of the injection micropipette cause different *θ*. The rotation moment *m* is decided by *θ* at this moment and should be optimized. Equation (10) is derived by taking the derivative of Equation (9):

(10) $$m^{\prime }=-F_{I}*\sqrt{R^{2}-R_{H\_in}{}^{2}}*\cos(\theta -\beta )$$

According to Equation (10), when *θ* is equal to *β*, the rotation moment *m* is at its maximum value. According to the parallel line theorem, the poking direction of the injection micropipette is parallel to the *X* axis at this moment. It is the optimal poking direction, in which the angular orientation of the contact point between the injection micropipette and the cell *θ* is equal to *β*. Meanwhile, the rotation force *F_I_* should be parallel to the *X* axis.

**Figure 4:**
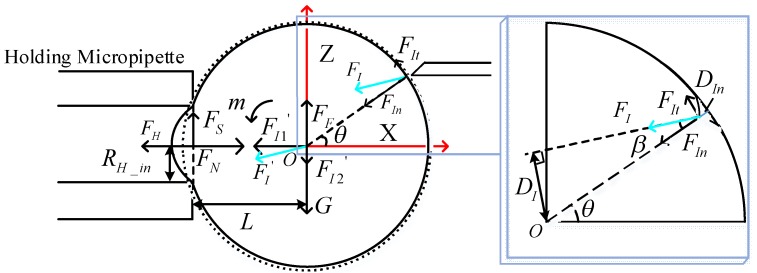
Force analysis of oocyte in the *X-Z* plane.

We can calculate the optimal direction of the injection micropipette in the revolving course as the same as the rotation course. Figure 4 depicts the schematic of forces in the revolving course. Due to gravity *G* and buoyancy *F_F_* along the center of oocyte *O*, they do not produce the rotation moment *m* at *O* in the *X*-*Z* plane. Therefore, the rotation moment *m* at *O* in the *X*-*Z* plane is the same as it is in the *X*-*Y* plane.

When *θ* is equal to *β*, the rotation moment *m* is at its maximum value. It is the optimal poking direction, in which the rotation force *F_I_* is parallel to the *X* axis.

### 2.2. The Strategy of Robotic Cell Rotation Based on Optimal Poking Direction 

**Figure 5:**
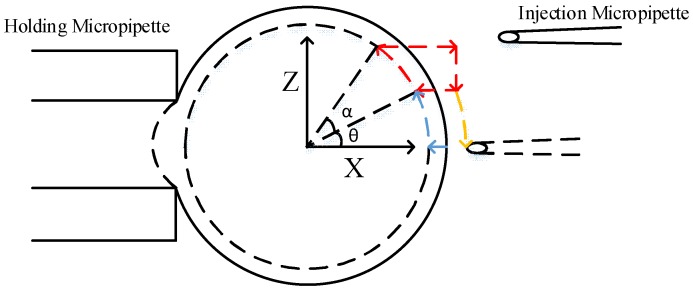
Rotation strategy of the first stage (front view).

The oocyte is required to be rotated to a desired orientation where the polar body is visible. The desired orientation can be determined by the operator. It is necessary to use a strategy of robotic cell rotation based on the optimal poking direction of the injection micropipette. In order to overcome slippage between the injection micropipette and the oocyte, the micropipette should maintain a constant poking depth in the revolving course. Therefore, the revolving trajectory was designed as a circular motion. Figure 5 illustrates the trajectory of the revolving course. In this course, the injection micropipette moved in the *X-Z* plane and rotated around the *Y* axis. The injection micropipette could not revolve around the cell normally clockwise when it lay in the first quadrant (the first stage) [22], therefore, it moved counterclockwise. First, the injection micropipette poked to a certain depth and moved around the track in *θ*, shown in blue in Figure 5. Then, the injection micropipette moved along in a circular motion in *α* on the track shown in red in Figure 5, until the polar body was observed; the small amplitude strategy was applied in this process. The small amplitude strategy realized the function of real-time revolving, which divided the 60° amplitude into steps of 10°. In order to rotate the polar body to the focal plane exactly before biological manipulation, we applied the second trajectory to the revolving course, and the amplitude of each step was reduced to 5° when the polar body was nearly observed. At the same time, the injection micropipette was moved to the focal plane and then to the rotation course when the polar body was visible.

**Figure 6:**
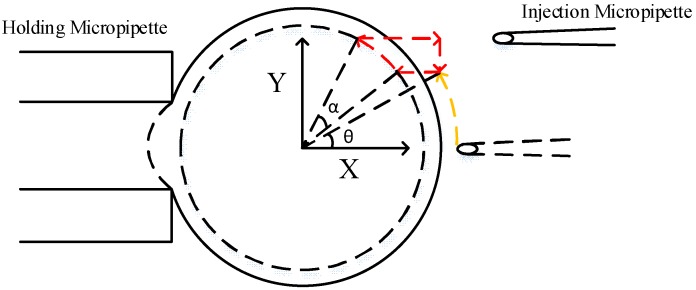
Rotation strategy of the second stage (vertical view).

Figure 6 shows the motion trajectory of the rotation course. In this course, the injection micropipette moved in the *X-Y* plane and rotated around the *Z* axis. The small amplitude strategy was also designed to make it more exact for the rotation course to realize the same real-time function as the revolving course. First, the injection micropipette was moved counterclockwise, along the track in *θ* shown in yellow in Figure 6. Then the injection micropipette poked to a certain depth and moved along in a circular motion in *α* along the track shown in red in Figure 6 until the polar body was rotated to the desired position. To improve precision in the rotation course, the second trajectory of the injection micropipette was used, the same as the revolving course. The experiment reduced each step to 5° when the polar body was close to the desired position (such as 11 o’clock).

## 3. Results

### 3.1. Experimental Facilities

**Figure 7:**
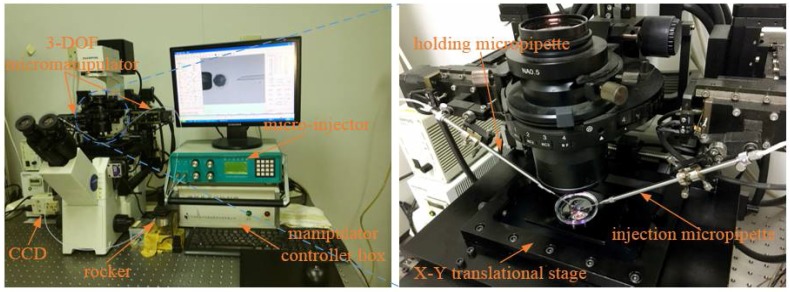
The NK-XMR160 micromanipulation system.

The robotic cell rotation experiment was performed by the NK-XMR160 micromanipulation system (NK, Tianjin, China), which was developed by our laboratory and is shown in Figure 7. It consists of the following facilities: An inverted microscope (Olympus CK-40, Chroma Technology Corp, Bellows Falls, VT, USA) forms the basic experimental facility. A charge-coupled device (CCD) (Panasonic W-V-460, Panasonic Corporation, Osaka, Japan) was installed on the experimental facility to gather microscopic images at 20 frames/s, to be processed by a host computer (i5, 2.8-GHz, 4GB RAM). The motorized *X-Y* translational stage (with a travel range of 100 mm × 100 mm, repeatability of ±1 μm, and maximum speed of 2 mm/s) was installed to position the hybrid substrate, which is placed in a 35 mm Petri dish. A pair of 3-DOF micromanipulators are used to position the injection micropipette (φ20 μm) and the holding micropipette (φ120 μm). The maximum speed of 3-DOF micromanipulators is 1 mm/s, with a repeatability of ±1 μm and a travel range of 50 mm. A pneumatic syringe was fixed on the 3-DOF micromanipulators to provide negative holding pressure (at a range of 0–3 kPa and resolution of 10 Pa) and positive injection pressure (at a range of 0–200 kPa and resolution of 10 Pa). The pressure data acquisition and motion control of the platform and micromanipulators are processed by the host computer.

### 3.2. Experimental Process

In order to verify the feasibility of the robotic cell rotation strategy proposed in this paper, we did the experiments on an NK-XMR160 micromanipulation system. The injection micropipette was assembled on the micromanipulator and moved by controlling the micromanipulator. The success rate of rotating the oocyte to the desired position, standard deviation, and average completion time were used to evaluate the strategy. Completion time was the total rotation time during which the cell was rotated from the initial position to the desired angular position. Standard deviation was calculated from total rotation time.

A total of 30 mature porcine oocytes, divided into two groups (groups A and B), were used to perform robotic cell rotation with different moving speeds of the injection micropipette starting from the optimal poking direction. The injection micropipette started from the same initial position in all experiments of optimal poking direction. The goal of revolving was to adjust the cell to the desired orientation (11 o’clock). Through many previous experiments, we found that the poking position of the injection micropipette *θ* should equal 30°. In summary, the poking position of the contact point between the injection micropipette and the *X* axis *θ* is 30° and the injection micropipette should be parallel to the *X* axis. The experimental process was organized as follows:

(1) Adjust the injection micropipette to the optimal direction.
(2) Set the radius of the circular motion according to the cell radius.
(3) Set *θ* to 30° (*θ* is alterable, and the operator can modify it according to the experimental requirements).
(4) Move the injection micropipette counterclockwise in the *X-Z* plane and rotate it around the *Y* axis. When the polar body is visible, the operator manually stops the first stage and proceeds to the second stage.
(5) The injection micropipette is moved counterclockwise in the *X-Y* plane and rotated around the *Z* axis. When the polar body is rotated to the desired orientation, the operator manually stops the second stage.
(6) The injection micropipette is automatically withdrawn to a suitable position and the experiment is over.

We did some experiments on nonoptimal poking direction to compare with the experiments on optimal poking direction. Another 30 mature porcine oocytes, also divided into two groups (groups C and D), were used to perform the same robotic cell rotation as in the experiment on optimal poking direction. The experimental process was similar, except for (1). In the experiments on nonoptimal poking direction, the injection micropipette was not parallel to the *X* axis. The injection micropipette started from the same initial position as in the experiments on nonoptimal poking direction.

### 3.3. Experimental Results

**Figure 8:**
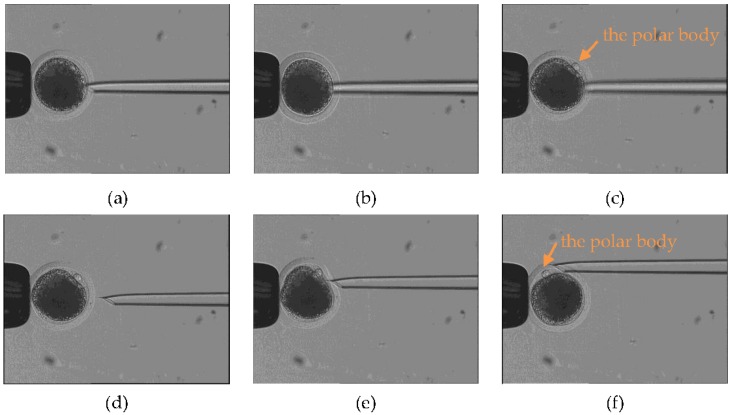
Experiment on optimal poking direction, showing the process of revolving. (**a**) The strategy’s starting point, as shown by the red track in Figure 5; (**b**) the revolving course in progress; (**c**) the strategy’s endpoint, as shown by the red track in Figure 5, with the polar body visible. (**d**–**f**) The process of rotation when the polar body is in the focal zone (second stage). (**d**) The injection micropipette moves to the starting point of the rotation course; (**e**) the rotation course in progress; (**f**) the strategy’s endpoint, where the polar body has been rotated to the desired position.

**Figure 9:**
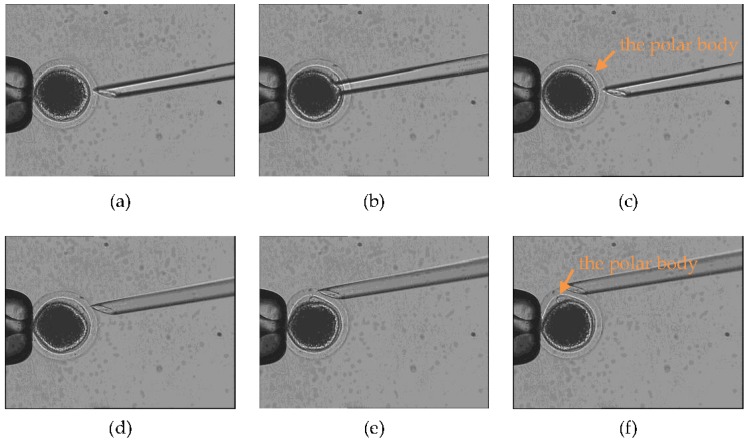
Experiment on nonoptimal poking direction. (**a**–**c**) The process of revolving (first stage). (**d**–**f**) The process of rotation when the polar body is in the focal zone (second stage). The injection micropipette is not parallel to the *X* axis.

Figure 8 shows the revolving course (first stage) and rotation course (second stage), in which the injection micropipette started from the optimal poking direction. As shown in Figure 8a–c, the injection micropipette was not in the focal zone when it moved in the red track shown in Figure 5. Figure 8d–f show the process of rotation when the polar body was in the focal zone (second stage), where it was required to be rotated to the desired orientation. Figure 9 shows the revolving course and rotation course, in which the injection micropipette started from the nonoptimal poking direction and was not parallel to the *X* axis.

For group A of oocytes, the moving speed of the injection micropipette was 100 μm/s, and the system rotated 14 oocytes to the desired position successfully, with a success rate of 93.3%. The single failure was due to the obvious elliptical shape of the oocyte. Because the motion trajectory was standard circular motion, the radius of the oocyte was estimated to determine the injection micropipette motion. In order to avoid failure in future experiments, we should try our best to improve the circle radius detection algorithm. For group B of oocytes, the moving speed of the injection micropipette was 200 μm/s and 13 oocytes were successfully operated, hence the success rate was 86.7%. The reason for failure was that the cell membrane was too soft. The softer the cell membrane is, the more viscous it becomes. The stickiness of the cell membrane increased the resistance force between the oocyte and the holding micropipette. In order to choose good elastic cells, in future research we should try to add the step of measuring the elasticity of the cell membrane.

For group C of oocytes, when the moving speed of the injection micropipette was 100 μm/s, the system only rotated 13 oocytes to the desired position successfully, for a success rate of 86.7%. For group D of oocytes, when the moving speed of the injection micropipette was 200 μm/s, 12 oocytes were successfully operated, and the success rate was only 80%.

For groups A and B (experiment on optimal poking direction), when the moving speed of the injection micropipette was 100 μm/s, the average completion time was 23.6 s, which was 5 s less than in [21]. With increased moving speed, the average completion time was 17.8 s. Slippage could be effectively avoided, and the success rate of rotating the oocyte was up to 86.7%. When the moving speed of the injection micropipette was 100 μm/s, standard deviation was 5.5. As the moving speed was increased to 200 μm/s, standard deviation remained at 5.7, effectively proving the consistency of this proposed strategy. For groups C and D (experiment on nonoptimal poking direction), when the moving speed of the injection micropipette was 100 μm/s, average completion time was 30.4 s and standard deviation was 5.9. When the moving speed of the injection micropipette was 200 μm/s, average completion time was 22.9 s and standard deviation was 6.2. Comparing the results of the optimal poking experiment and the nonoptimal poking experiment strongly proves the effectiveness of the strategy, in which we had the biggest moment of force. The proposed strategy effectively reduced the completion time. The experimental results are shown in Table 1.

## 4. Discussion

Three-dimensional orientation is needed for biological manipulation. In this paper, in order to find the optimal poking direction of the injection micropipette, we analyzed the force of cell rotation. A strategy of robotic cell rotation based on optimal poking direction was proposed, so that the specific structure of the cell can be moved to the desired orientation before biological manipulation. Experimental results show that when the speed of the injection micropipette was up to 100 μm/s, the average completion time was 23.6 s and the success rate was 93.3%. Meanwhile, the circular motion effectively overcame slippage between the injection micropipette and the oocyte. With increased moving speed of the injection micropipette, slippage could be effectively avoided and the success rate of the rotating oocyte was maintained up to 86.7%. Moreover, the experiment on nonoptimal poking direction was designed to compare with the experiment on optimal poking direction. The experimental results demonstrate that this strategy had the advantages of real-time operation, slip-resistance, and high efficiency. Future work will focus on automatic cell rotation, which adds a visual detection program to the proposed strategy, replacing the manual work of detecting the polar body.

## Figures and Tables

**Table 1:** The relationship of average completion time, standard deviation, and success rate with the moving speed of the injection micropipette.

Index		Speed	
		100 μm/s	200 μm/s
Optimal poking direction	Average completion time	23.6 s	17.8 s
	Standard deviation	5.5	5.7
	Success rate	93.3%	86.7%
Nonoptimal poking direction	Average completion time	30.4 s	22.9 s
	Standard deviation	5.9	6.2
	Success rate	86.7%	80%
